# Wellbeing and Social Network Characteristics in Rural Communities: Findings from a Cohort in Social Housing in Cornwall, United Kingdom

**DOI:** 10.1007/s42413-022-00167-5

**Published:** 2022-05-19

**Authors:** Emily Long, Sebastian Stevens, Raluca Topciu, Andrew James Williams, Timothy James Taylor, Karyn Morrissey

**Affiliations:** 1 MRC/CSO Social and Public Health Sciences Unit, University of Glasgow, Glasgow, UK; 2 The European Centre for the Environment and Human Health, University of Exeter, Truro, UK; 3 Washington Singer Laboratories, University of Exeter, Exeter, UK; 4 School of Medicine, University of St Andrews, Fife, UK

**Keywords:** Social cohesion, Social network, Mental wellbeing, Health, Social housing, Social capital

## Abstract

The mental wellbeing of those living in resource poor and rural localities is a public health priority. Despite evidence of a link between social networks and mental wellbeing, little is known about this relationship in the context of rural and resource poor environments. The current study uses novel social network methodology to investigate the extent to which social network size and composition is related to mental wellbeing in a social housing community in rural England. Data come from 88 individuals living in social housing in Cornwall. These participants are part of a larger study of 329 social housing households surveyed in 2017 and 2018. Mental wellbeing was measured by the Short Warwick-Edinburgh Mental Wellbeing Scale (SWEMWBS). A series of multivariable linear regression models were used to test associations between social network characteristics and mental wellbeing. Social network size was significantly associated with the SWEMWBS (b = 0.39, p < 0.01), such that individuals with larger networks reported better mental wellbeing, but after controlling for community social cohesion, this effect dissipated. Neither gender composition or talking with network members about health and wellbeing were significantly associated with the SWEMWBS. Findings suggest that both the quantity of social connections and perceptions of community cohesion are moderately associated with mental wellbeing in rural and resource poor localities. As such, efforts to improve mental wellbeing would benefit from targeting multiple aspects of social relationships, rather than focusing solely on increasing the size of individuals’ social networks.

## Background

Mental ill-health represents the largest single cause of disability within the UK (Mental Health Taskforce, 2016), with mental wellbeing increasingly recognised as a critical public health concern. This has worsened during the Covid-19 pandemic (Mental Health Foundation, 2017; Pierce et al., 2020). Interventions to improve mental wellbeing are encouraged to take place-based approaches that allow mental wellbeing to be understood through the lens of individual and wider environmental factors (Local Government Association, 2018; Public Health England, 2015). Associations between social relationships and a range of health outcomes are well established (Latkin & Knowlton, 2015; Montgomery et al., 2020), and growing evidence suggests that social networks (i.e., the characteristics of social connections surrounding an individual) are also associated with mental wellbeing (Fiori et al., 2006; Long et al., 2020; Sweet et al., 2018).

However, research into the dynamics between social networks and mental wellbeing has, and continues to be, typically focused on middle-class urban environments, neglecting important place-based variation (Albert et al., 1998). This is a particular concern when it is known that living in a resource poor area (i.e., an area of deprivation) is a common risk factor for poor mental wellbeing (Elliott, 2016). Furthermore, the geographic isolation inherent in rural communities suggests important distinctions from urban social contexts (Henning-Smith et al., 2018; Whitacre et al., 2017). Therefore, in order for research to effectively inform public health and policy efforts to improve mental wellbeing, it is critical to elucidate the relationship between social networks and mental wellbeing in rural and resource poor localities. As such, the current study examined associations between social network characteristics and the mental wellbeing of social housing residents in a deprived, rural area of England.

### The Rural Context

Rural communities face unique challenges to maintaining health and wellbeing, including aging populations, limited access to health care, and high levels of mental health stigma (Douthit et al., 2015; Mental Health Foundation, 2017; Public Health England, 2019). Rural areas also face specific challenges related to social isolation, including limited public transportation, digital access, and practical constraints on facilitating relationships between people in less densely populated areas (Henning-Smith et al., 2018; Whitacre et al., 2017). As a result, although overall mental health tends to be better in rural communities compared to urban settings (Elliott, 2016), the circumstances associated with poor mental health may function differently in the rural environment. Despite the acknowledged importance of social networks to an individual’s mental wellbeing (Fiori et al., 2006; Long et al., 2020; Sweet et al., 2018) and their health more broadly (Latkin & Knowlton, 2015; Montgomery et al., 2020), the lack of research examining these dynamics specifically in rural communities limits our understanding of how social networks may relate to mental wellbeing in rural settings.

Socioeconomic disadvantage is a well-established risk factor for poor mental wellbeing (World Health Organisation, 2014). Although individuals in rural communities typically exhibit better mental health than their urban counterparts (Elliott, 2016), important differences related to socioeconomic status remain. For example, typical examination of urban–rural differences in mental wellbeing using national statistics utilise broad-sweeping measures of rurality that can mask pockets of deprivation and poor wellbeing (Mental Health Foundation, 2017). Rural areas tend to be larger geographically and therefore include more diverse sub-populations when using standard geographical boundaries (Morrissey et al., 2008; Phillimore & Reading, 1992). Given the heterogeneous nature of rural areas, aggregate measures of health status are likely to be nonrepresentative and unreliable (Morrissey et al., 2008; Phillimore & Reading, 1992).

In response, this study seeks to overcome these limitations by focusing specifically on a rural and resource poor area. Given the unique social context of rural areas and the known vulnerabilities associated with deprivation, the current study employs a contextualised, place-based approach to investigate the relationship between social connections and mental health. By doing so, this study provides novel insights into (i) the social network characteristics of individuals living in rural and resource poor localities and (ii) how these network characteristics relate to mental wellbeing.

Specifically, the study focuses on three main characteristics of social networks potentially related to mental health. First, the study examined the extent to which social network size was associated with mental wellbeing. Based on previous findings from population-based data (Cornwell & Waite, 2009), we hypothesized that individuals with smaller social networks would report worse mental wellbeing. Second, the study examined associations between mental wellbeing and having people to talk with about health and wellbeing. Based on research demonstrating the importance of having people to talk with about personal topics (Latkin & Knowlton, 2015), we hypothesized that having a larger proportion of network members whom a respondent could speak with about health and wellbeing would be associated with better mental wellbeing. Lastly, we examined the extent to which the gender composition of networks was associated with mental wellbeing (Platt et al., 2014; Rice et al., 2012), hypothesizing that individuals with greater gender diversity within their networks would report better mental wellbeing.

## Methods

### Study Design and Participants

The study consists of 88 individuals from households participating in the Smartline Project (Smartline), 27% of the 329 households participating in the overall project. All households participating in the Smartline Project were contacted and invited to participate in the study. The Smartline Project focuses on residents of a local social housing provider from the towns and villages of Camborne, Pool, Illogan and Redruth in West Cornwall, UK. In the UK context, social housing is a form of low-income housing, where rent is lower than private tenancies, and the properties are owned by local government or non-profit organisations, such as housing associations. This area was chosen as it contains the highest concentration of social housing run by the partner housing association, as the project sought to understand the role of the community in participant health and wellbeing. A number of additional features of the study location contribute to the importance of studying social networks and their association to mental wellbeing. Firstly, Cornwall is located on a peninsula and only borders one other county in England. This area faces significant geographic barriers, being distant from major cities (with Plymouth and Exeter being at least 80 min travel away) and 100 miles from the nearest motorway. The Smartline study location, the Camborne, Pool, Illogan and Redruth area is a post-industrial mining area, and while proximal to the Cornish beaches that underpin Cornwall’s tourism industry, this area remains off the tourist trail.

Social network data was collected over winter 2018/2019 using an interview administered survey, conducted by a researcher via telephone at a time convenient to the participant. Participants were asked about their social network by listing the names and characteristics (e.g., gender) of up to eight individuals in their local area whom they meet and discuss matters important to them. Participants were also asked if they are able to talk to these nominated individuals about their health and wellbeing. All participants provided consent to participate in the study.

### Measures

#### Mental Wellbeing

Mental wellbeing was assessed during recruitment into the project using the Short Warwick-Edinburgh Mental Wellbeing Scale (SWEMWBS), a well-validated measure that covers both feeling and functioning aspects of mental wellbeing (Tennant et al., 2007). The scale consists of 7 items (e.g., ‘I’ve been feeling optimistic about the future’, ‘I’ve been feeling relaxed’), each measured on a 5-point scale, ranging from ‘none of the time’ to ‘all of the time’. Following standard protocol (Tennant et al., 2007), scores were summed, resulting in a variable ranging from 7–35, with higher scores indicating higher mental wellbeing.

#### Social Network Characteristics

Three social network variables were created from survey items. *Social network size* was measured by the total number of individuals the participant listed in their social network. The *gender similarity* of participant social networks was created using the E-I index (Krackhardt, & Stern, 1998), which provides a relative measure of similarity between an individual and their social connections based on a specified attribute (e.g., gender). The gender similarity variable ranges from -1 to 1, with -1 indicating that all ties were the same gender as the participant, and 1 indicating that no ties were the same gender. *Proportion of network with whom you discuss health and wellbeing* was calculated by the ratio of social ties participants indicated they could speak with about health and wellbeing versus the total number of social ties listed.

#### Control Variables

The current study accounted for a range of variables potentially associated with mental wellbeing, taken from the survey during recruitment into the whole Smartline project. *Age* was included and measured as a continuous variable. Education was measured by a variable ranging from 1 to 5, representing primary education through postgraduate university education. The Index of Multiple Deprivation (*IMD 2015)* and an 8-item measure of social cohesion developed by White et al. (2014), from Buckner's Neighbourhood Cohesion Scale (Buckner, 1998), were also included. The eight items measuring social cohesion relate to relationships with friends and neighbours, and activities such as visiting, helping in an emergency, borrowing and exchanging favours, with participants rating each statement from ‘strongly agree’ to ‘strongly disagree’ (5-point Likert scale).

Given evidence that pet ownership may improve mental wellbeing (Powell et al., 2019), a binary indicator of *pet ownership* was included. *Physical health-related quality of life* was measured with the widely-used and well-validated SF-12v2 Physical Component Summary (Jenkinson et al., 1993), which is composed of 12 items measuring eight domains of health outcomes (e.g., physical functioning, bodily pain) resulting in a variable which ranges from 0–100. A binary measure of whether the participant was *retired*, and a categorical measure of *household size* were also included.

### Statistical Analyses

Multivariable linear regression models were used to test the association between social network characteristics and mental wellbeing. In order to control for the known associations between sociodemographic characteristics and mental wellbeing (World Health Organisation, 2014), a stepwise modelling procedure was used. First, baseline univariable linear regression models were conducted to investigate the unadjusted association between each predictor variable and the SWEMWBS score (Model 1). Second, an experimental model using sociodemographic control variables and theoretically relevant predictors, but unadjusted for social network variables, was estimated (Model 2). Next, a social network model was conducted (Model 3), in which mental wellbeing was estimated based on the three network variables (e.g., network size, proportion of ties to same gender, proportion of ties discussing health and wellbeing), adjusted for the sociodemographic control variables, but excluding the experimental variables. The final model (Model 4) retained the statistically significant variables from the previous models, in addition to any variables with a p-value less than 0.2. The aim of this procedure was to produce the most parsimonious model and assess the extent to which variables remained significantly associated with mental wellbeing after adjusting for each set of potential predictors.

The sequential regression models were compared using the Root Mean Squared Error (RMSE) and the adjusted R-squared. All models were conducted using the ‘lm’ function within R. Item-level missingness was relatively low, with 1.12% missingness on mental wellbeing, and 0%—2.25% on all other variables.

## Results

Respondents were approximately 60 years old on average, and the sample was approximately 64% female. The sample used in this study, compared to the total Smartline Project sample, was slightly older on average, more likely to live in a neighbourhood classified in the 10% most deprived in England, and more likely to have only primary education. They were also more likely to be in smaller households and be retired. Average network size was approximately 5 people, and average SWEMWBS score was approximately 24 (range 7–35). See Table 1 for the full descriptive statistics of the sample and comparison to those who did not participate in the social network survey from the wider Smartline project.

**Table 1:** Participant characteristics

Characteristic		SNA participant (n = 86)	SNA non-participant (n = 239)	p-value
Sociodemographics				
Age (mean years ± SD)		59.7 ± 14.7	52.0 ± 17.9	< 0.01
Gender	Male	34.9% (n = 30)	29.7% (n = 71)	0.37
	Female	65.1% (n = 56)	70.3% (n = 168)	
10% most deprived neighbourhood*		69.8% (n = 60)	42.4% (n = 103)	< 0.01
Education	Pre-16	76.7% (n = 66)	61.8% (n = 147)	0.01
	Post-16	23.3% (n = 20)	38.2% (n = 91)	
Mental wellbeing (mean SWEMWBS ± SD)		24.1 ± 4.9	24.1 ± 5.5	0.95
Experimental variables				
Pet owners		61.6% (n = 53)	60.9% (n = 148)	0.91
Household size	1	47.7% (n = 41)	37.9% (n = 92)	0.04
	2	33.7% (n = 29)	28.8% (n = 70)	
	3 +	18.6% (n = 16)	33.3% (n = 81)	
Social cohesion (mean ± SD)		27.0 ± 6.0	26.8 ± 6.5	0.82
Physical health related quality of life (mean ± SD)		39.9 ± 13.5	41.0 ± 14.1	0.56
Retired		47.7% (n = 41)	28.0% (n = 68)	< 0.01
Social network variables				
Network size (median (IQR))		5 (4–8)	-	-
Gender homophily (median (IQR))		0.6 (0.3–1.0)	-	-
Proportion of network with whom you discuss health and wellbeing (median (IQR))		0.5 (0.3–0.9)		

^*^ Based on IMD. IQR – Interquartile range, SD – standard deviation

Results from the regression models demonstrated significant associations with mental health across a range of variables. Results from Model 1, which included individual associations between each predictor variable and the SWEMWBS score demonstrated significant associations for age (*b* = 0.10, 95% CI: 0.03–0.17), social cohesion (*b* = 0.21, 95% CI: 0.04–0.38), retirement (*b* = 3.18, 95% CI: 1.17–5.20), and social network size (*b* = 0.52, 95% CI: 0.06–0.98). Results from Model 2, which included effects related to sociodemographic variables and experimental variables only, indicated that increasing age (*b* = 0.14, 95% CI: 0.02–0.26) and higher social cohesion (*b* = 0.19, 95% CI: 0.02–0.36) were associated with better mental wellbeing. Results from Model 3 demonstrated significant associations between network size and mental wellbeing (*b* = 0.50, 95% CI: 0.03–0.97), such that larger social networks were related to better mental wellbeing, and age remained associated with wellbeing (*b* = 0.13, CI: 0.05–0.21). No evidence was found for the effect of gender composition or the discussion of health and wellbeing on mental wellbeing in this model. Finally, Model 4 found that none of the included variables remained significant, with the coefficients for social cohesion and social network size both being attenuated. The adjusted R^2^ indicates that Model 4 explained 24% of the variation in mental wellbeing (Table 2). The RMSE for Model 4 was the lowest of the included models, which suggested that it was an improvement in the fit of the model (Pham, 2019).

**Table 2:** Regression models of the associations with mental wellbeing

		Model 1	Model 2	Model 3	Model 4
		Unadjusted	Experimental model	Social network model	Final model
		Coef (95% CI)	Coef (95% CI)	Coef (95% CI)	Coef (95% CI)
Sociodemographics					
Age (years, centred)		**0.10 (0.03–0.17)**	**0.14 (0.02–0.26)**	**0.14 (0.06–0.22)**	0.10 (< -0.01–0.20)
Gender	Male	(ref)	(ref)	(ref)	(ref)
	Female	0.83 (-1.40–3.05)	1.05 (-1.22–3.33)	1.08 (-1.72–3.88)	0.94 (-1.13–3.01)
Not 10% most deprived neighbourhood*		-1.59 (-3.88–0.70)	-1.69 (-3.91–0.53)	-1.69 (-3.92–0.54)	-1.42 (-3.61–0.77)
Education	Pre-16	(ref)	(ref)	(ref)	-
	Post-16	-0.83 (-3.34–1.68)	0.48 (-2.03–2.98)	0.73 (-1.81–3.26)	-
Experimental variables					
Pet owners		-0.21 (-2.39–1.98)	0.34 (-1.84–2.51)	-	-
Household size	1	(ref)	(ref)	-	-
	2	-0.73 (-3.13–1.67)	-0.32 (-2.78–2.14)	-	-
	3 +	-0.85 (-3.77–2.06)	1.92 (-1.59–5.44)	-	-
Social cohesion (centred)		**0.21 (0.04–0.38)**	**0.19 (0.02–0.36)**	-	0.15 (-0.03–0.32)
Physical health related quality of life (centred)		0.27 (-0.05–0.11)	0.05 (-0.03–0.12)	-	-
Retired		**3.18 (1.17–5.20)**	1.10 (-1.95–4.15)	-	1.02 (-1.90–3.93)
Social network variables					
Network size		**0.52 (0.06–0.98)**	-	**0.49 (0.04–0.95)**	0.34 (-0.13–0.80)
Gender homophily		0.21 (-1.99–2.41)	-	0.07 (-2.76–2.89)	-
Proportion of network with whom you discuss health and wellbeing		-0.51 (-3.78–2.75)	-	1.21 (-2.03–4.46)	
Intercept		-	22.91 (20.05–25.77)	20.42 (15.71–25.14)	21.67 (18.25–25.10)
Adjusted R^2^			0.16	0.13	0.24

^*^ Based on IMD Bold = p <0.05. Coef = regression coefficient, 95% CI = 95% confidence interval

## Discussion

Mental wellbeing is a key priority area within the UK (Mental Health Foundation, 2017), and growing evidence suggests important links between social networks and mental wellbeing (Fiori et al., 2006; Long et al., 2020; Sweet et al., 2018). Given that living in a resource poor area is a known risk factor for poor mental wellbeing (Elliott, 2016), and the unique social challenges faced by rural communities (Douthit et al., 2015; Henning-Smith et al., 2018; Whitacre et al., 2017), this study sought to disentangle the associations between social network characteristics and mental wellbeing in a rural and resource-poor locality in England.

The study tested three primary patterns through which social networks may relate to mental wellbeing; social network size, gender similarity within networks, and the proportion of network members with whom a participant could discuss health or wellbeing. Social network size appears to be associated with mental wellbeing, but not quantity of ties alone, as when modelled alongside social cohesion, both variables lost their significance. The study found no evidence that network composition (i.e., gender similarity) or talking to network members about health and wellbeing related to mental wellbeing. Thus, this study suggests that the association between mental wellbeing and social networks in the study sample is more complicated than these mechanisms.

The findings moderately align with previous research using population-based data that demonstrated a protective effect of larger social networks (Cornwell & Waite, 2009). Specifically, the current findings suggest that the buffering effect of larger networks on mental wellbeing may extend to individuals in rural and resource-poor areas, but this effect is attenuated after perceptions of social cohesion are considered. Though previous research has demonstrated positive associations between having people to talk with about personal topics and mental health (Latkin & Knowlton, 2015), we found no evidence of this. Similarly, the current study did not find significant associations between gender similarity within networks and mental wellbeing, in contrast to previous work (Platt et al, 2014; Rice et al., 2012).

In addition to patterns related to social network characteristics, the study also found that individuals who were older, and those who reported higher levels of perceived social cohesion experienced better mental wellbeing, though these effects dissipated after controlling for social network characteristics. Though social cohesion has been previously linked to mental wellbeing (Williams et al., 2020), this association lost statistical significance once adjusted for social network size. It should be noted that the social networks elicited in the current study were specific to the local area, and it is possible that measures of networks that extend beyond the community may: 1) not dampen the effect of social cohesion, and 2) remain significantly associated with mental wellbeing even after accounting for social cohesion. Taken together, findings from the study suggest that social relationships are linked to mental wellbeing in rural areas, but both quantity (e.g., network size) and quality (e.g., social cohesion) of relationships are important.

Several limitations of the study must be recognised. First, the current sample size is relatively small; a restriction of the full study design. A larger number of participants would enable more complex analyses and more precision in the estimates. Second, participants were limited to naming at most eight members of their social network. More comprehensive documentation of social networks is needed to further explore the associations discovered, but is more time consuming for researcher and participant. Third, the study is cross-sectional in nature, precluding the analysis of changes to social networks and mental wellbeing over time. However, for the purposes of the current study, cross-sectional data were sufficient to assess the patterns through which social connections were associated with mental wellbeing. The limited sample restricts the number of potential control variables, as does the relatively homogeneous population – for example, we do not consider individual income or measures of wealth. Lastly, and perhaps most critically, the current study is limited by a very small number of characteristics gathered about each social connection (e.g., gender), and information on the connections between the social ties was not collected. Future research should aim to collect a more comprehensive set of data regarding participants’ social connections, which would allow for more detailed aspects of networks (e.g., network density) to be considered alongside the network characteristics featured in this study.

Despite these limitations, the study makes an important contribution to research on rural wellbeing by providing novel insight into the social network characteristics of individuals living in a rural and resource poor community, and begins to explore how these characteristics relate to mental wellbeing. Findings from the study suggest that those seeking to improve mental wellbeing in rural and resource poor settings should consider social network size when developing, rolling out, and evaluating interventions. Further, providing community space and activities that encourage social engagement (e.g., community hubs) may foster wider social connectivity and cohesion, and in turn, better mental wellbeing.

## Data Availability

An anonymised version of the dataset analysed during the current study is available upon request from the Smartline project repository: https://www.smartline.org.uk/data/

## Code Availability

Available upon request from EL.
